# Functional Outcomes of Knee Arthrodesis for Infected Total Knee Arthroplasty

**DOI:** 10.7759/cureus.46397

**Published:** 2023-10-03

**Authors:** Janet D Conway, Abhijith Annasamudram, Talal Abalkhail, Jalen H Tom, Robert P Farley, Martin Gesheff, Ahmed H Elhessy

**Affiliations:** 1 Orthopedics, International Center for Limb Lengthening, Rubin Institute of Advanced Orthopedics, Baltimore, USA; 2 Orthopaedics, King Faisal Specialist Hospital and Research Centre, Riyadh, SAU; 3 Orthopedic Surgery, University of Maryland, College Park, USA; 4 Orthopedics, LifeBridge Health, Baltimore, USA; 5 Medicine, University of Maryland School of Medicine, Baltimore, USA

**Keywords:** proms, tka revision, tka, pji, periprosthetic joint infection, knee arthrodesis

## Abstract

Introduction: As the occurrence of total knee arthroplasties (TKAs) is forecasted to continue rising, so too will the frequency of prosthetic joint infections (PJIs) and revision TKAs. Multiple revisions can result in an unreconstructible knee. In such instances, the knee may be salvaged through arthrodesis. We evaluated whether height, BMI, and age impacted patient-reported outcome measures (PROMs) in patients who underwent knee arthrodesis after revision TKA due to PJI.

Methods: We conducted a retrospective review of patients undergoing arthrodesis for an infected TKA at a dedicated orthopedic infection service from 2014 to 2022. Patient demographics and PROMs from 36-Item Short Form Survey (SF-36) and Knee Injury and Osteoarthritis Outcome Score (KOOS) questionnaires were collected. Correlation analysis was performed to determine if any association between height, BMI, and age was present with the various PROMs and sub-scores.

Results: Forty-four patients (19 males, 25 females) were included, with a mean follow-up of 48 months. Increases in height (>166 cm), BMI (>30), and age (>62 years) had a statistically significant negative impact on three SF-36 components: health changes (P = 0.016), physical functioning ability (P = 0.0096), and general health components (P = 0.0075).

Conclusion: Our results suggest that a knee arthrodesis is an acceptable option in patients with a persistent knee PJI with good functional PROMs and ambulatory status. Patients with shorter height, lower BMI, and younger age showed overall better outcomes. Knee arthrodesis can be an alternative option for amputation in patients with an infected TKA and provide good functional outcomes in selected patients.

## Introduction

Primary total knee arthroplasty (TKA) is a reputable procedure with reproducible results. Nevertheless, some patients achieve suboptimal results after undergoing TKA surgery and find that revision TKA is necessary. As the number of TKA procedures is forecasted to continue rising (increasing by 673% from 2005 to 2030), so too will the frequency of revisions (increasing by 601% in the same timeframe) [1].

Approximately 25% of all revision TKAs are related to prosthetic joint infection (PJI) [2]. Despite the evolution of antibiotics, there has been a steady growth in the virulent and resistant strains of bacteria over the past decade. The five-year mortality rate of PJI in the United States (US) has been reported to be higher than breast cancer, melanoma, Hodgkin’s lymphoma, and other common malignancies. [2] The cost of treating PJI in the US is substantial, with combined annual hospital costs approximated at over one billion dollars as of 2017, projected to double by 2030 [3].

Multiple revision surgeries due to PJI of the knee can result in an unreconstructable knee. Knee arthrodesis is a salvage option that can provide a stable weight-bearing limb and is the option that imparts superior function and ambulation when compared with above-knee amputation (AKA) [4]. In the current literature, the patient-reported outcome measures (PROMs) in patients who underwent knee fusion have been reported to be better than those who have undergone an AKA after an infected TKA. The reported cases of infected TKAs who underwent an arthrodesis ranged from 0.21% to 1.11% [5,6].

We aimed to assess patient-reported outcome measures after knee arthrodesis in individuals with infected and unreconstructible total knee arthroplasty. We also assessed whether patients' height, age, and BMI affected the PROMs in knee arthrodesis patients with infected TKAs.

## Materials and methods

A retrospective chart review was performed on a series of patients who underwent arthrodesis for an infected TKA, performed from January 2014 to January 2022 at a single center (Sinai Hospital of Baltimore) with a dedicated orthopedic infection service. Demographic data collected included sex, age, race, height, weight, BMI, Charlson Comorbidity Index (CCI), ambulation status, and functional and mental PROMs. Patients were excluded from this analysis if they underwent a subsequent knee replacement following arthrodesis, underwent a below- or above-knee amputation, had inadequate medical records (such as PROMs unavailable), or had less than six months of follow-up. An exemption status from our institutional review board (LifeBridge Health IRB) was received for this study.

**Figure 1:**
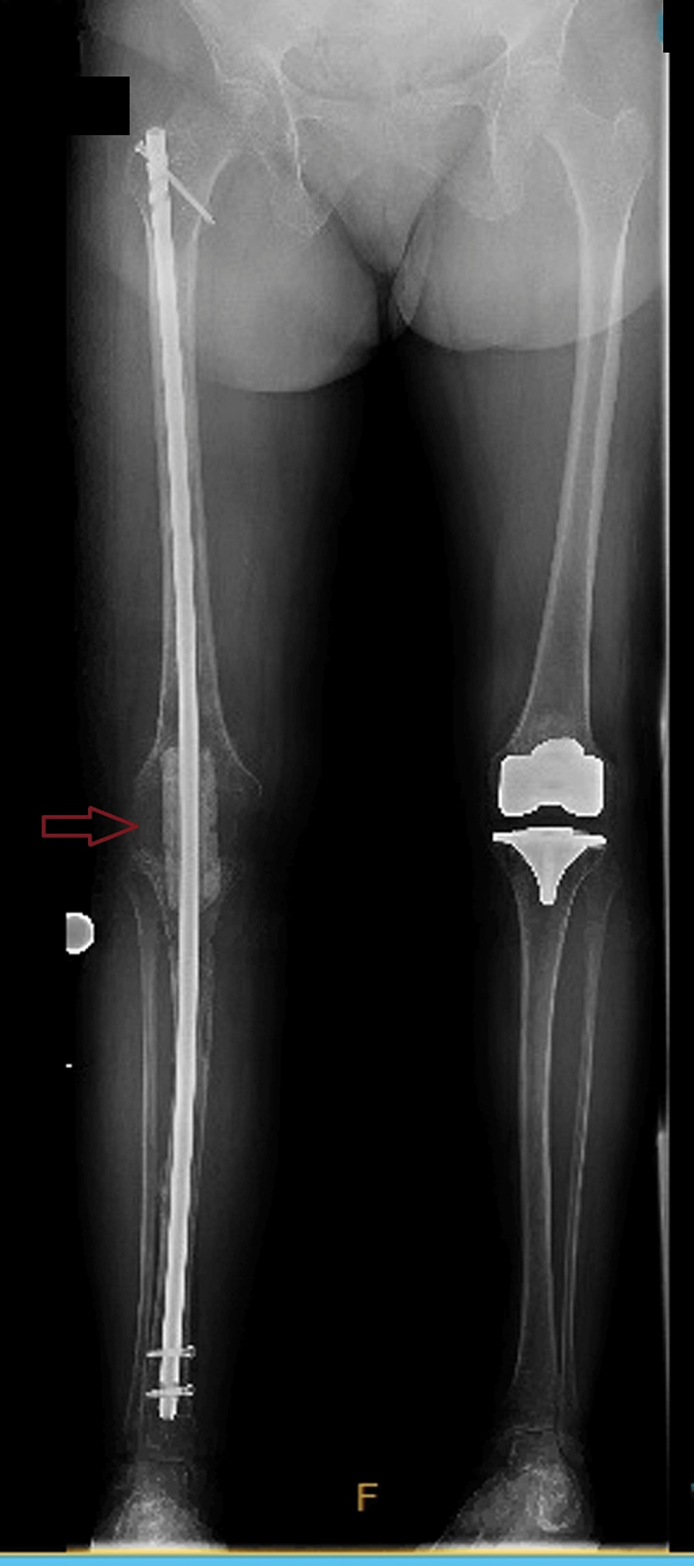
Long nail Anteroposterior plain radiograph showing the right knee long antibiotic-cement-coated intramedullary nail with the Palacos bone cement spacer block (arrow)

**Figure 2:**
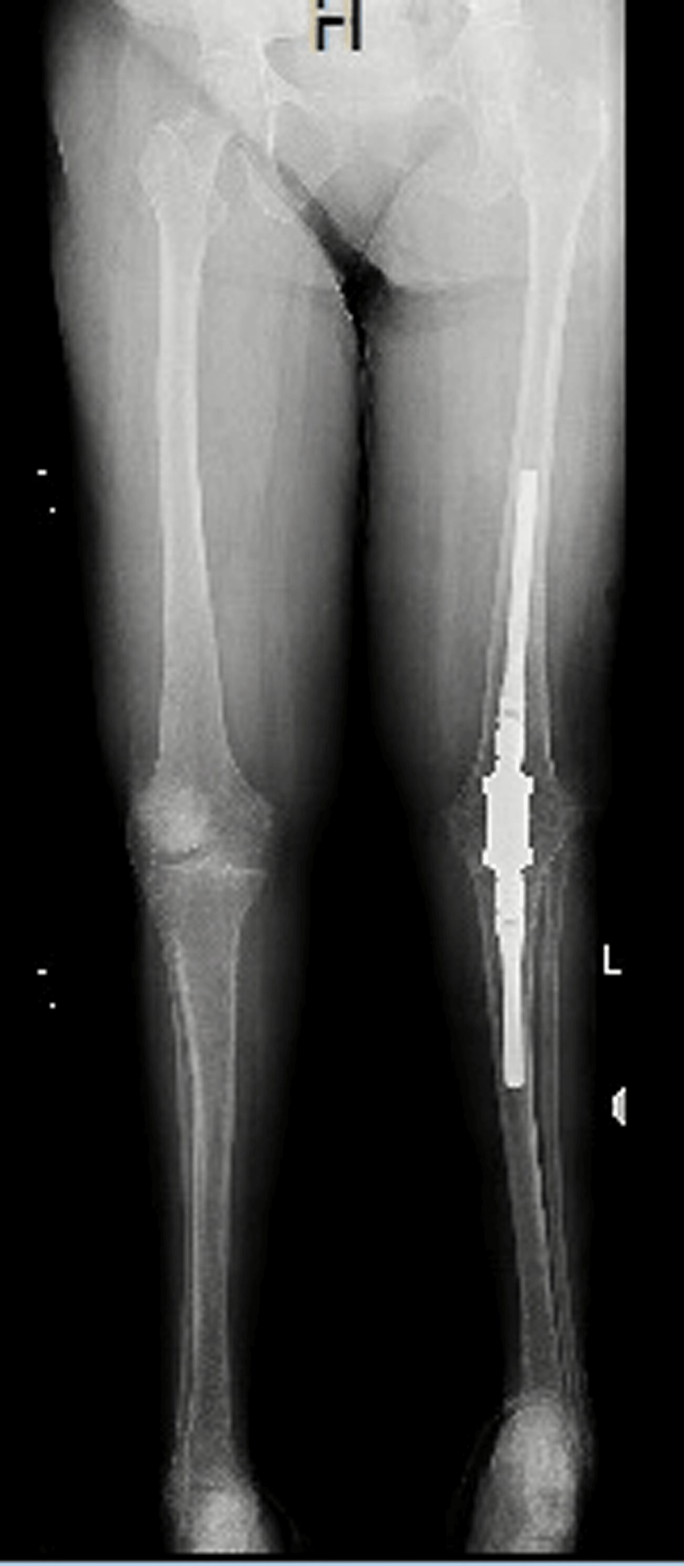
Short nail Anteroposterior plain radiograph showing the left knee short coupling nail with antibiotic-impregnated calcium sulfate injected into the canals before implant insertion

All knee arthrodesis surgeries were performed by a single surgeon, the first author, and consisted of either a long knee antibiotic-cement-coated intramedullary nail (ACCIN) or a short coupling nail with antibiotic-impregnated calcium sulfate injected into the canals before implant insertion (Figures 1, 2).

Palacos bone cement (Zimmer Biomet, Warsaw, IN) was used for the ACCINs, each pack containing 40 g of polymethyl methacrylate; 1 g of vancomycin and 3.6 g of tobramycin were added to each pack.

The 36-Item Short Form Survey (SF-36) and Knee Injury and Osteoarthritis Outcome Score (KOOS) survey responses were recorded to measure and analyze PROMs postoperatively. The SF-36 questionnaire consists of eight scales yielding two summary measures: physical and mental health. The physical health measurement scales are physical functioning (10 items), role-physical (four items), bodily pain (two items), and general health (five items). The mental health measurement scales are vitality (four items), social functioning (two items), role-emotional (three items), and mental health (five items). A total score of 100 indicates no symptoms and 0 indicates maximum symptoms. The KOOS evaluates both short- and long-term consequences of a knee injury. It comprises 42 items in five separately scored subscales: pain, other symptoms, function in daily living (activities of daily living, or ADL), function in sports and recreation (sport/rec), and knee-related quality of life [7]. Standardized answer options (five-point Likert scale) are given, and each question is assigned a score from 0 to 4. An overall score of 100 indicates no symptoms, and 0 indicates extreme symptoms. Surveys were collected from patients during outpatient visits or via phone calls by a research team member.

Based upon responses obtained from the SF-36 section regarding limitation of activities, we classified patients into three categories based on ambulatory status: home ambulators (patients ambulating at home only), community ambulators (patients who can ambulate a block or more away from home), and non-ambulators (wheelchair- or bed-bound).

Patient-reported outcome measures obtained from knee arthrodesis patients were compared to the normal US population as controls. The scores obtained from different components of the SF-36 questionnaire were compared with those of the general population from the data collected in 2012. This comparison evaluated the deviations and differences in PROM scores between knee arthrodesis patients and the general population.

To assess the effect of height, BMI, and age on function, patients were separated into groups and compared with the PROMs. Patients were filtered into one of the two groups that were dependent on a total height of ≤166 cm or >166 cm (Table 1).

**Table 1:** Test scores of patients grouped by height KOOS, Knee Injury and Osteoarthritis Outcome Score; SD, standard deviation; SF-36, 36-Item Short Form Survey

Scores	Height ≤166 cm (n = 23), mean ± SD	Height >166 cm (n = 21), mean ± SD	P value
KOOS	64.2 ± 17.8	59.2 ± 16.06	0.31
SF-36			
Emotional well-being	65.7 ± 21.6	62.5 ± 23.5	0.62
Energy/fatigue	46.9 ± 20.9	46.3 ± 21.8	0.91
General health	50.1 ± 21.1	52.9 ± 26.5	0.68
Health changes	66.7 ± 29.6	46.3 ± 26.3	0.016
Physical function	46.2 ± 34.2	32.2 ± 29.6	0.13
Role limitation due to emotional problems	55.1 ± 40.9	51.7 ± 47.5	0.79
Role limitation due to physical health	32.9 ± 36.8	24.5 ± 35.8	0.43
Social functioning	60.8 ± 27.9	57.9 ± 30.8	0.73

Similarly, two groups were created for BMI totaling either <30 or ≥30 (Table 2).

**Table 2:** Test scores of patients grouped by BMI BMI, body mass index; KOOS, Knee Injury and Osteoarthritis Outcome Score; SD, standard deviation; SF-36, 36-Item Short Form Survey

Scores	BMI <30 (n = 15), mean ± SD	BMI ≥30 (n = 29), mean ± SD	P value
KOOS	61.20 ± 14.61	62.91 ± 13.71	0.71
SF-36			
Emotional well-being	63.82 ± 19.82	64.68 ± 22.31	0.89
Energy/fatigue	48.32 ± 18.58	45.52 ± 21.18	0.66
General health	44.88 ± 19.24	55.34 ± 23.41	0.14
Health changes	67.33 ± 24.04	51.03 ± 29.50	0.07
Physical function	55.62 ± 34.10	30.01 ± 27.15	0.0096
Role limitation due to emotional problems	57.88 ± 38.69	51.29 ± 47.23	0.64
Role limitation due to physical health	28.85 ± 33.36	27.14 ± 37.25	0.88
Social functioning	54.81 ± 25.04	61.94 ± 30.41	0.44

All BMIs were documented on the day of the procedure. We arrived at the deciding factor of 166 cm for our height groups based on an investigation into the average heights of adult Americans of varying genders, ages, and races [8]. The deciding factor of 30 for our BMI groups was due to a score of ≥30, signifying obesity [9]. Lastly, we created two groups for patients aged ≤62 years and >62 years (Table 3). This age was chosen as our deciding factor for age groups because our population’s mean age at the time of the procedure was 62.3 years.

**Table 3:** Test scores of patients grouped by age KOOS, Knee Injury and Osteoarthritis Outcome Score; SD, standard deviation; SF-36, 36-Item Short Form Survey

Scores	Age ≤62 years (n = 19), mean ± SD	Age >62 years (n = 25), mean ± SD	P value
KOOS	62.1 ± 15.3	60.8 ± 20.5	0.81
SF-36			
Emotional well-being	65.9 ± 20.3	60.2 ± 26.7	0.42
Energy/fatigue	47.7 ± 20.3	45.6 ± 23.6	0.83
General health	57.8 ± 20.6	38.3 ± 25.4	0.0075
Health changes	54.5 ± 25.4	60.3 ± 37.4	0.52
Physical function	36.6 ± 32.3	32.4 ± 32.6	0.45
Role limitation due to emotional problems	55.8 ± 45.8	48.04 ± 40.5	0.57
Role limitation due to physical health	26.08 ± 35.9	34.16 ±37.3	0.48
Social functioning	64.09 ± 29.2	49.3 ± 27.2	0.1

After removing patient identifiers, the data was collated into Excel (Microsoft, Redmond, WA) for the initial review. Subsequent statistical analysis was performed using MedCalc statistical software, version 20.009 (MedCalc Software Ltd, Ostend, Belgium). Baseline demographics were summarized using means with standard deviations and percentages of the studied population. Descriptive statistics were calculated for continuous and categorical data using Student's t-test and Fisher's exact test. A P-value less than 0.05 was considered statistically significant. The CI was reported between −1 and 1, with 1 portraying a positive correlation. For comparison of subgroups and scores between healthy and study populations, an independent sample t-test of means was utilized (Tables 4, 5).

**Table 4:** Demographics and PROMs BMI, body mass index; KOOS, Knee Injury and Osteoarthritis Outcome Score; PROM, patient-reported outcome measure; SD, standard deviation; SF-36, 36-Item Short Form Survey

Variable	Mean	±SD
Current age (years)	66.9	13.1
Age at the procedure (years)	62.3	12.7
Follow-up time (months)	48	35
BMI	33.8	9.3
Height (cm)	164	13.06
Weight (kg)	90.7	20.3
KOOS	63	13.9
SF-36		
Emotional well-being	64.3	21.3
Energy/fatigue	46.4	20.1
General health	51.8	22.4
Health changes	56.5	28.5
Physical functioning	38	31.7
Role limitation due to emotional problems	53.3	44.1
Role limitation due to physical health	27.7	35.5
Social functioning	59.5	28.6
Pain	47.8	28.02

**Table 5:** Correlation coefficient and significance of height, BMI, and weight with PROMs BMI, body mass index; KOOS, Knee Injury and Osteoarthritis Outcome Score; PROM, patient-reported outcome measure; SD, standard deviation; SF-36, 36-Item Short Form Survey

Test and PROM		Height	BMI	Weight
KOOS	Correlation coefficient	-0.099	0.048	-0.026
	Significance level (P)	0.521	0.75	0.867
SF-36				
Emotional well-being	Correlation coefficient	-0.11	-0.037	0.355
	Significance level (P)	0.475	0.809	0.018
Energy/fatigue	Correlation coefficient	-0.112	-0.072	-0.172
	Significance level (P)	0.467	0.642	0.265
General health	Correlation coefficient	-0.052	-0.044	-0.056
	Significance level (P)	0.738	0.778	0.716
Health changes	Correlation coefficient	0.24	-0.166	-0.05
	Significance level (P)	0.117	0.281	0.747
Physical functioning	Correlation coefficient	0.073	-0.269	-0.259
	Significance level (P)	0.636	0.0776	0.089
Role limitation due to emotional problems	Correlation coefficient	-0.047	-0.143	-0.21
	Significance level (P)	0.763	0.354	0.171
Role limitation due to physical health	Correlation coefficient	0.046	-0.107	-0.064
	Significance level (P)	0.767	0.488	0.68
Social functioning	Correlation coefficient	-0.025	-0.107	-0.156
	Significance level (P)	0.873	0.489	0.312
Pain	Correlation coefficient	0.071	0.238	-0.206
	Significance level (P)	0.644	0.119	0.178

## Results

A total of 51 patients who underwent arthrodesis for an infected TKA were initially identified and met our inclusion criteria. Of these, four patients were deceased and three patients were lost to follow-up; thus, 44 patients had follow-up data of more than six months and ultimately comprised our study population. Twenty-eight of these patients had >24 months' follow-up. The mean follow-up period was 48 months (range, 6-122 months). There were 19 (43.2%) males and 25 (56.8%) females. Among them, 26 (59%) were Caucasians, 17 (38.6%) African Americans, and 1 (2.3%) was Asian. The mean age at the time of the procedure was 62.3 years (SD ±12.7 years), mean BMI was 33.8 (SD ±9.3), mean height was 164.8 cm (SD ±13.06 cm) and the mean weight was 90.7 kg (SD ± 20.3 kg).

The most common comorbidities in our patient population were type 2 diabetes mellitus and hypertension. The mean CCI score was 1.13 (range, 0-5). Thirty patients received a long ACCIN, whereas 14 received a short antibiotic-coated coupling nail fusion. Twenty-eight patients were community ambulatory, and 16 were home ambulators at the end of the treatment in our study.

The mean and SD of the various SF-36 components in the normal US population have been cataloged in a 2012 study by Maglinte et al. [10]. We tallied the means ± SDs for the SF-36 PROMs from our cohort and compared them with the results of Maglinte et al. (Table 6).

**Table 6:** Comparison of SF-36 scores with the general population PROM, patient-reported outcome measure; SD, standard deviation; SF-36, 36-Item Short Form Survey *General population numbers derived from the study by Maglinte et al. [10]

SF-36 PROM	Mean ± SD, our study population		Mean ± SD, general population*		P value
Emotional well-being	64.3	21.3	54.2	13.2	<0.01
Energy/fatigue	46.4	20.1	53.7	15.3	<0.01
General health	51.8	22.4	50.1	16.8	0.5
Physical functioning	38	31.7	50.6	14.4	<0.01
Role limitation due to emotional problems	53.3	44.1	51.4	13.2	0.36
Role limitation due to physical health	27.7	35.5	49.4	14.7	<0.01
Social functioning	59.5	28.6	51.3	13.9	<0.01
Pain	47.8	28.02	50.6	16.2	0.25

We discovered statistically significant differences existed in five categories when comparing our study cohort and the general population. These five SF-36 components were emotional well-being, energy/fatigue, physical functioning, role limitation due to physical health, and social functioning. Three SF-36 score components were higher in the knee fusion group than in the general population. These were general health, role limitation due to emotional problems, and pain.

The SF-36 and KOOS results were compared among the two groups that we established for each of our three focuses of height, BMI, and age. There were 23 patients in our shorter group and 21 in the taller group. A statistical significance between the two height groups was found in the health changes component of the SF-36 (P = 0.016); the shorter group had a significantly better score (Table 1). Our BMI groups comprised 15 patients with BMI <30 and 29 with BMI ≥30. A statistical significance between the two BMI groups was found in the physical function component of the SF-36 (P = 0.0096); the <30 BMI group had a significantly better score (Table 2). The patients were divided into two groups based on their age: a group with patients who were 62 years old or younger (19 patients) and another group with patients older than 62 years (25 patients). The younger age group had a higher score in the general health component of SF-36 (Table 3). KOOS scores did not reveal any statistical significance for any of our groups. Among height, BMI, and age, the KOOS P values were 0.31, 0.71, and 0.81, respectively.

## Discussion

The mean SF-36 and KOOS scores obtained in this study suggest that knee arthrodesis is considerably a good functional solution for PJI secondary to TKA. Furthermore, when testing the hypothesis that advanced ages, taller heights, and higher BMIs correlate with worse PROMs, we found this to be true in overall score trends from the SF-36 and KOOS, but only noted statistical significance in one component of the SF-36 for each of our three categories. Moreover, the statistically significant component differed amongst the three categories. We can conclude that, statistically, patients aged <62 years, <166 cm tall, and with a BMI <30 had better scores, when compared to the rest of the studied cohort.

We compared the PROMs of knee fusion in our study population with the control group and found no statistically significant difference for three SF-36 score components of general health, role limitation due to emotional problems, and pain. Yet, the remaining five components of the SF-36 had significantly different scores between the general population and our group of arthrodesis patients. The better scores for the general population were expected due to additional energy expenditure when living with knee arthrodesis and the logistical challenges of functioning with a fused knee during activities of daily living. All patients in our study were ambulatory on an uninfected fused knee after a significant period of non-ambulatory status.

There is no consensus on a single “best” procedure for PJI management. Procedures for managing a knee PJI may include a two-stage TKA procedure, knee fusion, and AKA. Gomez et al. studied 504 patients with chronic PJI and found that approximately 20% of patients never underwent second-stage reimplantation [11]. Meehan et al. reported that patients aged <50 years are more prone to have a knee PJI than older patients [12]. Studies also indicate that one-stage procedures are reasonable options for treating an infected revision TKA [13].

Arthrodesis of the knee can eradicate the infection and successfully provide a stable leg for ambulation [14,15]. Knee arthrodesis procedures have been performed successfully as a salvage procedure for treating osteoarthritis since before TKA surgery existed [16]. Patients receiving knee fusions for recurrent PJIs after TKA are reported as having better functional outcomes than AKA, as Chen et al. reported in 2012 [4]. Twenty patients who underwent knee fusion for recurrent PJI found increased ambulation among knee fusion patients postoperatively, and they scored better on SF-12 when compared with AKA patients [4]. The alternative option of AKA does not allow for a reliable way to weight bear, particularly in elderly and low-demand patients [17,18]. Studies have indicated an increased energy expenditure in patients who underwent AKA surgery compared with those who underwent knee arthrodesis. The energy needed to ambulate on an AKA was reported as approximately 0.20 ml/kg/min; for a fused knee joint, it was 0.16 ml/kg/min [17,18].

In a 2020 study by Brown et al., 16 of the 17 patients with chronic knee PJI underwent successful knee fusion with an intramedullary antegrade nail [19]. They concluded that knee fusion was a suitable salvage treatment method for carefully selected patients with complex recurrent PJIs [15,19,20]. Barton et al. studied 12 patients treated with intramedullary knee fusion following knee PJI. The PROMs were assessed using the SF-12 and Oxford Knee Score after 53 months of follow-up and there were no significant difference in outcome scores following knee arthrodesis compared to the revision TKA [14].

A study was conducted by Klinger et al. including 20 patients comparing single- and two-stage knee fusion [21]. They reported that patients with two-stage fusion had good fusion rates and scored better on the SF-36 score components compared to the single-stage study population. They concluded that arthrodesis of the knee was a satisfactory salvage procedure following a failed TKA and provided reliable expectations for a stable, painless limb in high-functioning patients, preserving their ability to ambulate [21].

Robinson et al. in a recent article documented functional scores following knee arthrodesis for infected TKA [22]. They described 20 successful fusions and reported functional scores for 11 patients. Their average KOOS symptom score was 73.1. These scores compare positively with our study, which had an average KOOS score of 62.36. The Robinson et al. study had a small pool of patients and a variety of arthrodesis methods, including external fixation [22]. Our study included two lengths of intramedullary nails, allowing immediate postoperative weight bearing. This early weight bearing may have contributed to the good functional scores in our patients. Also, our study population consisted of 44 patients, whereas Robinson et al. reported on 20 patients [22].

Our study is not without limitations. The patient population in our study had a wide range of postoperative follow-up periods. The duration of recording postoperative functional scores was not uniform. Also, preoperative baseline KOOS and SF-36 PROMs were not obtained; these would have helped because a percentage of change could have been evaluated. Our study represents a large number of knee fusions performed using ACCINs from a single institute, which is a strength of this study.

Arthrodesis is a viable alternative for limb salvage in chronic infective conditions of the knee since it provides a functioning limb aiding in the patient's mobilization. While AKA may be perceived as a last resort, especially when complications are present with respect to soft tissue and the bony components, arthrodesis remains a conservative solution that must be considered first, particularly while using a long ACCIN or a short antibiotic-coated coupling nail. Our study indicates that knee arthrodesis is a functional procedure secondary to knee PJI, and the patients uniformly maintained the ability to ambulate independently and self-reported good functional scores and quality of life [23]. With this information, surgeons can counsel patients to maintain their independent ambulatory status with PROMs comparable to the normal healthy population, favorably in the shorter, non-obese, and younger age groups, yet further studies are encouraged to compare the PROMs between knee arthrodesis and other surgical options. In summary, knee arthrodesis can be a viable alternative to amputation in infected TKA populations in certain conditions.

## Conclusions

Our results suggest that knee arthrodesis can be a viable option for the treatment of persistent TKA PJI. The surgeon's experience and appropriate patient selection remain crucial prior to performing this procedure in this patient population. Further studies comparing the functional outcomes of knee arthrodesis and other treatment modalities for TKA PJI are required.

## References

[1] Kurtz S, Ong K, Lau E, Mowat F, Halpern M (2007). Projections of primary and revision hip and knee arthroplasty in the United States from 2005 to 2030. J Bone Joint Surg Am.

[2] Kurtz SM, Lau EC, Son MS, Chang ET, Zimmerli W, Parvizi J (2018). Are we winning or losing the battle with periprosthetic joint infection: trends in periprosthetic joint infection and mortality risk for the Medicare population. J Arthroplasty.

[3] Premkumar A, Kolin DA, Farley KX, Wilson JM, McLawhorn AS, Cross MB, Sculco PK (2021). Projected economic burden of periprosthetic joint infection of the hip and knee in the United States. J Arthroplasty.

[4] Chen AF, Kinback NC, Heyl AE, McClain EJ, Klatt BA (2012). Better function for fusions versus above-the-knee amputations for recurrent periprosthetic knee infection. Clin Orthop Relat Res.

[5] Wang CJ, Huang TW, Wang JW, Chen HS (2002). The often poor clinical outcome of infected total knee arthroplasty. J Arthroplasty.

[6] Blom AW, Brown J, Taylor AH, Pattison G, Whitehouse S, Bannister GC (2004). Infection after total knee arthroplasty. J Bone Joint Surg Br.

[7] Roos EM, Lohmander LS (2003). The Knee injury and Osteoarthritis Outcome Score (KOOS): from joint injury to osteoarthritis. Health Qual Life Outcomes.

[8] Fryar CD, Kruszon-Moran D, Gu Q, Ogden CL (2018). Mean body weight, height, waist circumference, and body mass index among adults: United States, 1999-2000 through 2015-2016. National Health Statistics Reports, no. 122. https://www.cdc.gov/nchs/data/nhsr/nhsr122-508.pdf.

[9] (2023). Defining adult overweight and obesity. https://www.cdc.gov/obesity/basics/adult-defining.html.

[10] Maglinte GA, Hays RD, Kaplan RM (2012). US general population norms for telephone administration of the SF-36v2. J Clin Epidemiol.

[11] Gomez MM, Tan TL, Manrique J, Deirmengian GK, Parvizi J (2015). The fate of spacers in the treatment of periprosthetic joint infection. J Bone Joint Surg Am.

[12] Meehan JP, Danielsen B, Kim SH, Jamali AA, White RH (2014). Younger age is associated with a higher risk of early periprosthetic joint infection and aseptic mechanical failure after total knee arthroplasty. J Bone Joint Surg Am.

[13] Massin P, Delory T, Lhotellier L (2016). Infection recurrence factors in one- and two-stage total knee prosthesis exchanges. Knee Surg Sports Traumatol Arthrosc.

[14] Barton TM, White SP, Mintowt-Czyz W, Porteous AJ, Newman JH (2008). A comparison of patient based outcome following knee arthrodesis for failed total knee arthroplasty and revision knee arthroplasty. Knee.

[15] Razii N, Abbas AM, Kakar R, Agarwal S, Morgan-Jones R (2016). Knee arthrodesis with a long intramedullary nail as limb salvage for complex periprosthetic infections. Eur J Orthop Surg Traumatol.

[16] Charnley J, Baker SL (1952). Compression arthrodesis of the knee: a clinical and histological study. J Bone Joint Surg Br.

[17] Waters RL, Perry J, Antonelli D, Hislop H (1976). Energy cost of walking of amputees: the influence of level of amputation. J Bone Joint Surg Am.

[18] Conway JD, Mont MA, Bezwada HP (2004). Arthrodesis of the knee. J Bone Joint Surg Am.

[19] Brown NM, Balkissoon R, Saltzman BM, Haughom B, Li J, Levine B, Sporer S (2020). Knee arthrodesis with an intramedullary antegrade rod as a salvage procedure for the chronically infected total knee arthroplasty. J Am Acad Orthop Surg Glob Res Rev.

[20] Conway JD, Elhessy AH, Galiboglu S, Patel N, Gesheff MG (2022). Efficacy of infection eradication in antibiotic cement-coated intramedullary nails for fracture-related infections, nonunions, and fusions. Antibiotics (Basel).

[21] Klinger HM, Spahn G, Schultz W, Baums MH (2006). Arthrodesis of the knee after failed infected total knee arthroplasty. Knee Surg Sports Traumatol Arthrosc.

[22] Robinson M, Piponov HI, Ormseth A, Helder CW, Schwartz B, Gonzalez MH (2018). Knee arthrodesis outcomes after infected total knee arthroplasty and failure of two-stage revision with an antibiotic cement spacer. J Am Acad Orthop Surg Glob Res Rev.

[23] Asghar A, Naaz S, Narayan RK, Kumar A (2021). Does the prevalence of ossified fabella vary in knee osteoarthritis and age-related degeneration? A meta-analysis of about 11,000 knees. Cureus.

